# Gallbladder Volvulus in an Elderly Patient: A Case Report and Review of Literature

**DOI:** 10.7759/cureus.45167

**Published:** 2023-09-13

**Authors:** Belén Matías-García, Carolina Sainz-Azara, Fernando Mendoza-Moreno, Manuel Díez-Alonso, Alberto Gutiérrez-Calvo

**Affiliations:** 1 General Surgery, Príncipe de Asturias Teaching Hospital, Alcalá de Henares, ESP; 2 Radiology, Príncipe de Asturias Teaching Hospital, Alcalá de Henares, ESP

**Keywords:** abdominal pain, floating gallbladder, acute cholecystitis, cholecystectomy, gallbladder volvulus

## Abstract

Gallbladder volvulus is an uncommon cause of acute cholecystitis that results from the rotation of the gallbladder about its mesentery along the axis of the cystic pedicle. We present the case of an 87-year-old woman with acute abdominal pain in the right upper quadrant that began two days prior with no additional symptoms. The physical examination showed a large abdominal eventration on the right side and tenderness. A CT scan showed a distended gallbladder outside its liver bed and with a twist on its cystic pedicle, suggesting a gallbladder volvulus. Abdominal ultrasonography complementary revealed the gallbladder outside its vesicular fossa with incipient changes of acute cholecystitis but without evidence of gallstones. With the diagnosis of acute abdomen secondary to gallbladder volvulus, an emergency cholecystectomy was performed. The exact etiology of the gallbladder volvulus is unknown, although anatomical variants that predispose to its appearance have been described. The clinical presentation is similar to acute cholecystitis, so preoperative diagnosis can be challenging for both surgeons and radiologists and is often misdiagnosed. This is one of the few cases diagnosed with preoperative imaging techniques. Once diagnosed, the appropriate treatment is emergency cholecystectomy. Early diagnosis and surgical treatment are important before it progresses to necrosis, perforation, biliary peritonitis, and hemodynamic instability. Gallbladder volvulus is an uncommon condition. A high index of suspicion is required because the preoperative diagnosis is unusual. Once the diagnosis is established, the treatment is fundamentally surgical.

## Introduction

Acute cholecystitis is one of the most frequent causes of acute surgical abdominal pain. Essentially, its origin is secondary to gallstones or infection, but there are other pathologies that can produce it. Gallbladder volvulus was first described in 1898 as a "floating gallbladder" by Wendel in a 23-year-old pregnant woman [1]. The finding of a gallbladder volvulus in an acute abdomen scene is an uncommon entity, with fewer than 600 cases described in the literature [2]. In the last 5 years, less than 50 cases have been described.

Gallbladder volvulus is defined as the rotation of the gallbladder on its mesentery along the axis of the cystic pedicle [3,4]. The incidence is higher in elderly women between 60 and 80 years of age, while in men it is more frequent in children than in adults [2,5,6]. It has an incidence of one in 365,520 hospital admissions. This represents approximately 1.5% of all cholecystectomies performed for acute cholecystitis [7].

The presence of gallstones is considered incidental with a rate of less than 30%, while cholelithiasis has been identified as a major culprit in most gallbladder diseases [8]. The clinical presentation mimics acute cholecystitis, and there are no specific preoperative symptoms, so the diagnosis is generally made intraoperatively [9]. However, there are radiological characteristics that suggest a preoperative diagnosis of gallbladder volvulus [10]. In patients with a preoperative diagnosis of gallbladder volvulus, early surgical treatment is indicated [5]. Delayed surgery is directly related to an increase in mortality of approximately 6%.

The aim of the study is to report an uncommon case of gallbladder volvulus in an elderly female diagnosed preoperatively and provide a review of the literature on this entity, analyzing the main risk factors for a proper and early diagnosis and management.

## Case presentation

An 87-year-old woman presented with acute abdominal pain located in the right upper quadrant that began two days prior with no additional symptoms. Medical history included chronic obstructive pulmonary disease and eventration secondary to rectocele surgery. In the emergency, the patient was hypertensive without tachycardia. The physical examination showed a large abdominal eventration in the pararectal right line, non-reducible pain, and palpation pain. Laboratory test results revealed a white cell count of 17.6 103/μl (neutrophils were 4.33 103/μL), while liver and pancreatic function tests were within normal limits. Chest and abdomen radiographs were performed without relevant findings.

**Figure 1:**
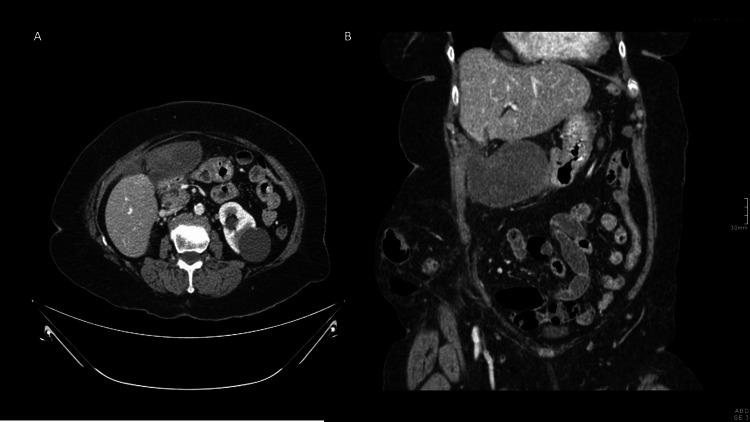
(A) CT scan findings showing a complete twist of artery and cystic duct; (B) CT scan showing a horizontalized gallbladder outside its anatomical fossa.

On suspicion of incarcerated eventration, a computerized tomography (CT) scan was performed. It showed a distended gallbladder outside its gallbladder fossa and with a twist on its vascular pedicle, suggesting a gallbladder volvulus (Figure 1).

**Figure 2:**
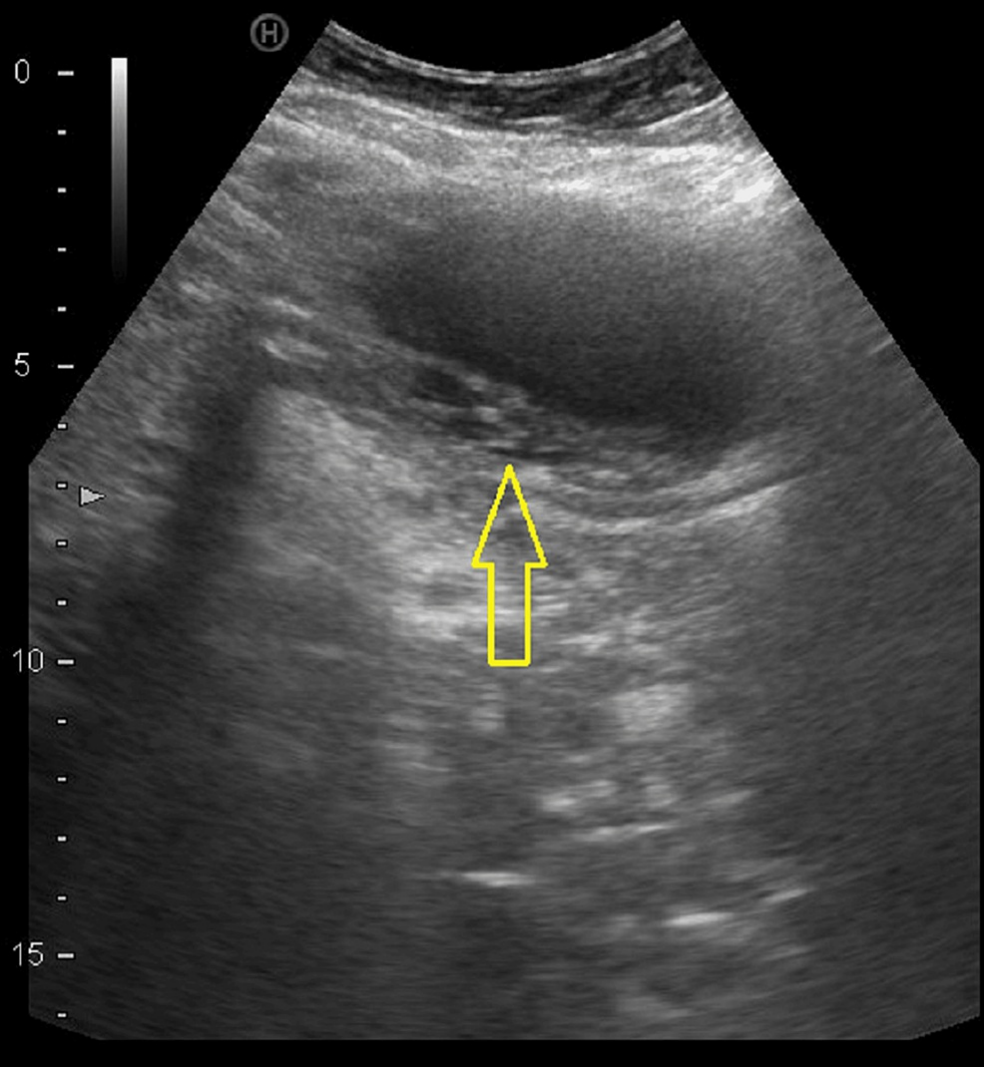
Ultrasonography findings that suggest acute cholecystitis (echogenic membranes inside gallbladder and wall thickening >3 mm).

An abdominal ultrasonography that was complementary showed that the gallbladder was outside of its vesicular fossa and showed incipient changes of acute cholecystitis, but there was no evidence of gallstones. Linear echogenic membranes were found (Figure 2).

**Figure 3:**
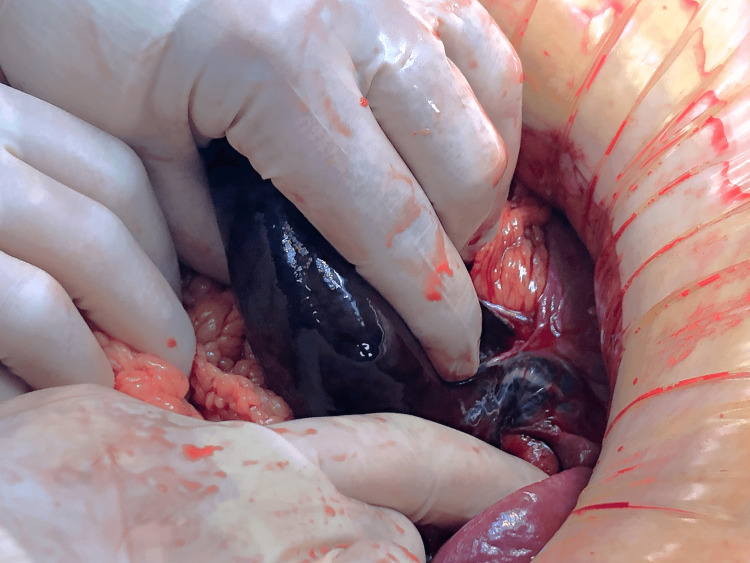
Necrotic gallbladder twisted at its pedicle with two 360° turns.

With the diagnosis of acute abdomen secondary to gallbladder volvulus and gangrenous cholecystitis, the patient was operated on as an emergency three hours after admission to the emergency room. An exploratory laparoscopy was performed, showing a gallbladder outside its fossa and with gangrene secondary to the torsion of the vascular pedicle with two 360º turns. The gallbladder was not perforated. There was free fluid around the gallbladder and subphrenic space. The procedure was converted to an open cholecystectomy due to technical difficulties encountered at laparoscopy due to huge abdominal eventration (Figure 3).

**Figure 4:**
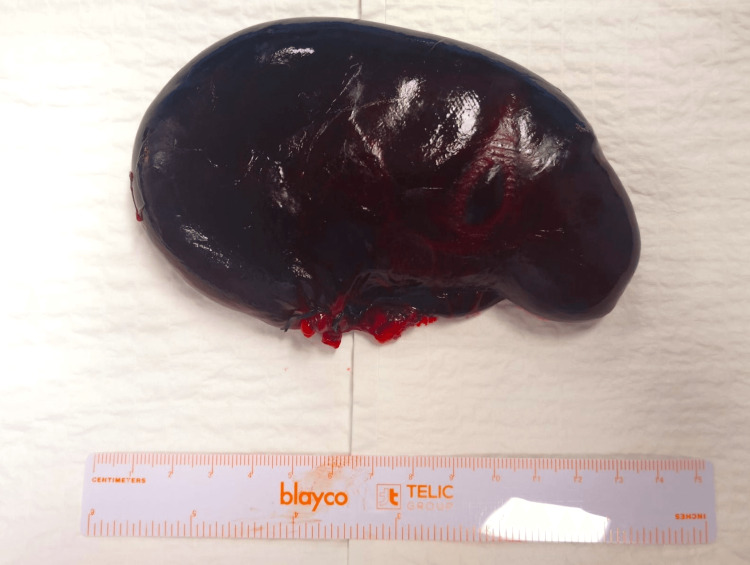
Anatomical piece of gangrenous gallbladder.

After identifying the bile duct, gallbladder devolvulation was performed, and the cystic artery and cystic duct were selectively ligated and transected. The gallbladder was disconnected from the hepatic hilium (Figure 4).

Abdominal cavity irrigation and a drainage tube were placed in the surgical wound. No other ischemic lesions were observed in the rest of the bowel or colon. The abdominal wall was repaired with no mesh placement. The patient was extubated after surgery without incident, not requiring admission to the intensive care unit.

**Figure 5:**
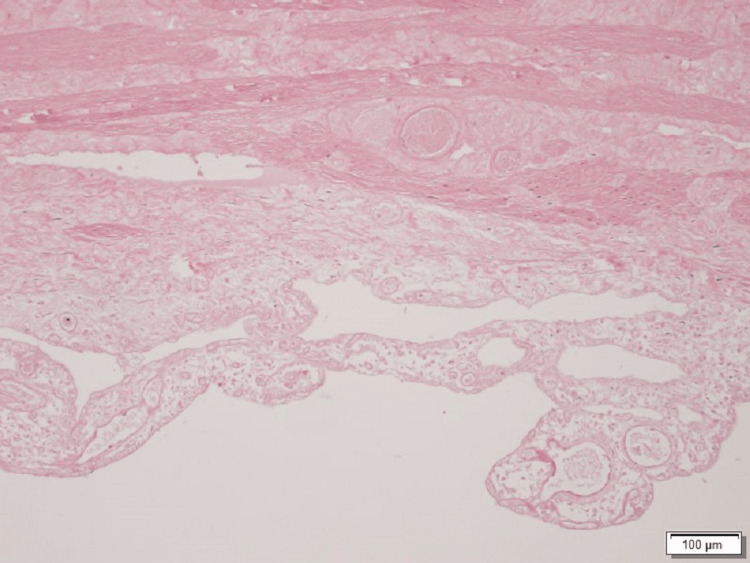
Histological section of gallbladder shows transmural necrosis, inflammatory infiltrate, vascular congestion and areas of bleeding (staining with hematoxylin-eosin).

The patient had an uncomplicated postoperative course, with good oral tolerance and staying afebrile. She was discharged on the sixth postoperative day; there were no readmissions, and the outpatient follow-up was uneventful after six months. Histopathological findings revealed a necrotic hemorrhagic gallbladder with no gallstones, according to acute gangrenous cholecystitis (Figure 5).

## Discussion

Gallbladder volvulus was first described in 1898 by Wendel as a "floating gallbladder" [1]. It is an uncommon disease defined as the rotation of the gallbladder on its mesentery along the axis of the cystic pedicle, with subsequent interruption of the vascular and biliary flow [3,4]. The reported incidence is approximately 1 in 365,000 hospital admissions [2,5]. It seems to have a bimodal distribution in terms of age. There is a group in the paediatric age range (0-18 years old) where it is more frequent in men, with a female/male ratio of 1:2.5 [10]. There is a second group of elderly patients (70-100 years old) where the incidence is higher in women, with a female/male ratio of 4:1 [10].

The exact aetiology of the gallbladder volvulus is unknown, although anatomical variants that predispose to its appearance have been described [3,4,10]. It is thought that there should be a congenital abnormality in gallbladder attachment, resulting in a hypermobile gallbladder [2,3,4]. According to Gross classification, a mesentery of variable length connects the liver with the gallbladder and the cystic duct (type A) or only with the cystic duct (type B) [3,11]. In our patient, the gallbladder volvulus adopted a type B configuration. Other uncommon anatomical variations are a hypermobile gallbladder due to a hypermobile liver secondary to the absence of coronary and triangular ligaments or a cystic duct with an aberrant origin in the right hepatic duct constituting a very narrow cystic pedicle [3]. These conditions are unusual and undetectable preoperatively.

Other reported non-congenital anatomical factors correlated with a higher incidence of torsion among the elderly population are elastic tissue and liver atrophy, resulting in the release of the gallbladder [9,11,12]. It is possible that significant weight loss with low visceral fat contributes to the hypermobility of the gallbladder [2,4]. Other factors described are spinal deformities like kyphosis, which places the gallbladder in a different position, and cystic artery hardening due to arteriosclerosis [3,9,11]. However, in addition to an anatomical predisposition, a twisting trigger is necessary. It has been postulated that the peristaltic movements of the stomach could be the triggers of a turn in a clockwise manner, while the peristalsis of the transverse colon would cause a counter-clockwise torsion [3,9]. Furthermore, the extent of torsion measured in degrees can be classified as incomplete, in which rotation is <180°, or complete, in which rotation is >180° [9,10]. Reilly et al., in a review of 327 articles, found no significant relationship between the direction of torsion and whether it was complete or incomplete [10].

The clinical presentation is similar to acute cholecystitis, so preoperative diagnosis can be challenging for both surgeons and radiologists and is often misdiagnosed [9,11,12]. It is reported that up to 10% of patients are diagnosed on imaging prior to surgery [13,14].

The main symptomatology consists of acute and intense abdominal pain in the right upper quadrant, associated with nausea and vomiting. However, due to the hypermobility of the gallbladder, the pain may be located in the middle or right lower quadrant [3]. Therefore, a high level of suspicion is essential. On physical examination, there may be a palpable abdominal mass in the absence of jaundice [3,11]. Lau et al. described a triad that suggests a gallbladder volvulus, but there are no specific preoperative symptoms [7,13,15].

The analytical findings show an acute inflammatory process with leukocytosis and left deviation without a clear alteration of liver enzymes [13,15]. Another peculiar clinical finding is the absence of clinical or serological improvement with the appropriate medical and antibiotic support for an infectious pathology [4]. Therefore, we must suspect a gallbladder volvulus in an elderly woman who is thin and kyphotic or has chronic lung disease with clinical features of acute cholecystitis and does not improve on supportive treatment and antibiotics [3,11,14].

The diagnosis is usually made intraoperatively, but since the early 1990s, preoperative diagnosis has been increasing due to improvements in medical imaging techniques [10,13]. Up to 26% of preoperative diagnoses have been reported [16,17]. Several ultrasound findings have been described that suggest a gallbladder volvulus: the location of the gallbladder outside its fossa, a twisted pedicle, a horizontalized gallbladder, and the absence of blood flow on Doppler ultrasonography [2,3,10]. Other findings include those typical of cholecystitis, such as a distended gallbladder with a thickened wall and pericholecystic fluid [3,11,14]. Cholelithiasis is only present in about 24% of patients [2,4,9]. Therefore, the presence of ultrasound signs suggestive of cholecystitis in the absence of cholelithiasis can suggest the diagnosis of gallbladder volvulus as an alternative [3,4,9]. CT findings are mainly the beak sign, as a result of the transition of the distended gallbladder to a fulcrum point in the gallbladder hilum, and the whirl sign, which represents the rotation of the pedicle [3,14,18]. Other findings are a massively distended gallbladder, pericholecystic fluid, and a gallbladder outside its anatomical fossa. Gallbladder torsion has been reported to show a ‘hot rim’ image on hydroxy-iminodiacetic acid (HIDA) scans as a result of the accumulation of the radioisotope within the gallbladder [2,10,11,19]. Although the use of magnetic resonance imaging is limited by its lower availability and the longer duration of the exploration, it could be useful if there are doubts after ultrasound and CT, and it provides more information in cases of wall necrosis or intramural haemorrhage [13].

Once diagnosed, the appropriate treatment is emergency cholecystectomy. Early diagnosis and surgical treatment are important before it progresses to necrosis, perforation, biliary peritonitis, and hemodynamic instability [4,5]. Cholecystectomy can be performed using a laparoscopic or open technique, but it can usually be treated with a minimally invasive approach [5,16]. Calot’s triangle may be distorted due to the abnormal position of the structures [11,13], so devolvulation prior to resection with careful dissection of the bile duct is recommended to avoid iatrogenic injury [2,3,9]. However, Price and DiMarco claim that detorsion in the state of necrosis can lead to toxin release secondary to reperfusion, ultimately leading to systemic effects [4].

The prognosis is excellent if an early cholecystectomy is performed [9]. However, if surgical intervention is delayed, the mortality rate of the gallbladder volvulus is estimated at 6%, especially since it is more common in the elderly population with multiple medical comorbidities, in which the initial management is usually conservative [3,10,11]. Non-operative management or percutaneous drainage are not indicated in the management of gallbladder volvulus because they do not resolve the cause [16].

## Conclusions

Gallbladder volvulus is an uncommon condition that must be considered in the differential diagnosis of acute cholecystitis in daily practice, as early surgery is preferable to calculous acute cholecystitis. A high index of suspicion is required because the preoperative diagnosis is unusual. Some risk factors and radiological findings, such as age, gender, or absence of blood flow in Doppler ultrasound, could suggest gallbladder volvulus. Once the diagnosis is established, the treatment is fundamentally surgical. Emergency cholecystectomy is the treatment of choice to prevent its progression to necrosis, perforation, and biliary peritonitis. Gallbladder volvulus is an acute surgical condition that is often misdiagnosed, which could increase its morbidity and mortality.
